# Vascular Inflammation Is Associated with Loss of Aquaporin 1 Expression on Endothelial Cells and Increased Fluid Leakage in SARS-CoV-2 Infected Golden Syrian Hamsters

**DOI:** 10.3390/v13040639

**Published:** 2021-04-08

**Authors:** Lisa Allnoch, Georg Beythien, Eva Leitzen, Kathrin Becker, Franz-Josef Kaup, Stephanie Stanelle-Bertram, Berfin Schaumburg, Nancy Mounogou Kouassi, Sebastian Beck, Martin Zickler, Vanessa Herder, Gülsah Gabriel, Wolfgang Baumgärtner

**Affiliations:** 1 Department of Pathology, University of Veterinary Medicine Hannover, 30559 Hannover, Germany; lisa.allnoch@tiho-hannover.de (L.A.); georg.beythien@tiho-hannover.de (G.B.); eva.leitzen@tiho-hannover.de (E.L.); kathrin.becker@tiho-hannover.de (K.B.); fkaup@gwdg.de (F.-J.K.); vanessa.herder@tiho-hannover.de (V.H.); 2 Department for Viral Zoonoses-One Health, Heinrich Pette Institute, Leibniz Institute for Experimental Virology, 20251 Hamburg, Germany; stephanie.stanelle-bertram@leibniz-hpi.de (S.S.-B.); berfin.schaumburg@leibniz-hpi.de (B.S.); nancy.mounogou@leibniz-hpi.de (N.M.K.); Sebastian.beck@leibniz-hpi.de (S.B.); martin.zickler@leibniz-hpi.de (M.Z.); guelsah.gabriel@leibniz-hpi.de (G.G.); 3 Institute for Virology, University for Veterinary Medicine Hannover, 30559 Hannover, Germany

**Keywords:** vasculitis, vasculopathy, SARS-CoV-2, COVID-19, aquaporin 1, hamster, perivascular edema, endothelium

## Abstract

Vascular changes represent a characteristic feature of severe acute respiratory syndrome coronavirus-2 (SARS-CoV-2) infection leading to a breakdown of the vascular barrier and subsequent edema formation. The aim of this study was to provide a detailed characterization of the vascular alterations during SARS-CoV-2 infection and to evaluate the impaired vascular integrity. Groups of ten golden Syrian hamsters were infected intranasally with SARS-CoV-2 or phosphate-buffered saline (mock infection). Necropsies were performed at 1, 3, 6, and 14 days post-infection (dpi). Lung samples were investigated using hematoxylin and eosin, alcian blue, immunohistochemistry targeting aquaporin 1, CD3, CD204, CD31, laminin, myeloperoxidase, SARS-CoV-2 nucleoprotein, and transmission electron microscopy. SARS-CoV-2 infected animals showed endothelial hypertrophy, endothelialitis, and vasculitis. Inflammation mainly consisted of macrophages and lower numbers of T-lymphocytes and neutrophils/heterophils infiltrating the vascular walls as well as the perivascular region at 3 and 6 dpi. Affected vessels showed edema formation in association with loss of aquaporin 1 on endothelial cells. In addition, an ultrastructural investigation revealed disruption of the endothelium. Summarized, the presented findings indicate that loss of aquaporin 1 entails the loss of intercellular junctions resulting in paracellular leakage of edema as a key pathogenic mechanism in SARS-CoV-2 triggered pulmonary lesions.

## 1. Introduction

Since its discovery in 2019 in Wuhan, China, severe acute respiratory syndrome coronavirus-2 (SARS-CoV-2) has emerged as the cause of the coronavirus disease 2019 (COVID-19) pandemic, challenging health care systems worldwide [1,2]. SARS-CoV-2 is a betacoronavirus that elicits a broad clinical spectrum ranging from asymptomatic or mild forms to severe or even fatal disease [3]. A total of 30% of the SARS-CoV-2 infected patients suffer from a severe clinical course with alveolar and vascular damage resulting in the activation of coagulation pathways and a consecutive disseminated intravascular coagulation [2,4,5].

Alveolar damage represents a rather nonspecific feature of pulmonary disease and can be attributed to a variety of respiratory viruses such as Middle East respiratory syndrome coronavirus (MERS-CoV), SARS-CoV, and influenza A viruses. However, vascular changes during SARS-CoV-2 infections including microangiopathy with disruption of intercellular junctions are presumed to represent a characteristic pattern in COVID-19 [5,6,7]. Damage of the respiratory vasculature comprises vasculitis, endothelialitis, and severe endothelial injury which finally results in a breakdown of the vascular barrier and subsequent edema [2]. Even though there are studies describing vascular changes at distinct time points during COVID-19, a detailed and continuous characterization and description of the vascular alterations with consecutive edema formation is still missing [5,8,9]. Increased vascular permeability with subsequent emission of fluid observed in severely affected lungs is assumed to be the result of several interrelating and simultaneously occurring mechanisms [2]. Examinations of lungs from patients who died from COVID-19 indicate that the recruitment of inflammatory cells either represents a direct result of viral infection or a solely immune-mediated feature, which may induce diffuse endothelial inflammation with dysfunction and apoptosis [2,5,8].

Endothelial cells represent a major component of the blood–air barrier. They play an essential role in blood vessel formation and ensure tissue homeostasis as well as vascular integrity [2,10]. The endothelial barrier function is maintained by intercellular junctions, namely adherens junctions and tight junctions, regulating, inter alia, the extravasation of plasma and macromolecules [11]. A directed and rapid transport of large amounts of water is indispensable for the maintenance of tissue homeostasis [10]. Therefore, aquaporins (AQPs) form water channels on the surface of endothelial cells and ensure a bidirectional and transcellular transport of water along an osmotic gradient generated by ions and solutes [10,12]. Mammalians possess 13 aquaporins, 4 of them (AQPs 1, 3, 4, and 5) located in the respiratory system. Aquaporin 1 (AQP1) is the only aquaporin expressed within the plasma membranes of microvascular endothelial cells [13,14]. Under physiological conditions, AQP1 is responsible for transcellular fluid transport, angiogenesis, and endothelial cell migration [10]. Furthermore, studies on different lung disorders support the assumption that AQP1 water channels play a role in various lung diseases [15]. Upregulation of AQP1 could be observed in acute lung injury and pulmonary fibrosis, whereas a downregulation was reported for acute respiratory distress syndrome, asthma, and adenoviral infections [12,16,17,18]. However, the exact role of aquaporins in human lung disease is not completely understood [12,15].

The aim of our investigation was to provide a detailed characterization of the vasculopathy during SARS-CoV-2 infection including an evaluation of the impaired vascular integrity. Therefore, we investigated and detailed the amount as well as the phenotype of contributing immune cells within different vascular compartments at different time points using the well-established golden Syrian hamster model [9,19,20,21,22,23,24,25]. Moreover, pathological changes were quantified and characterized via assessment of edema formation, ultrastructural endothelial integrity, and loss of the integral membrane protein AQP1.

## 2. Materials and Methods

### 2.1. Animal Experiments

Animal experiments were performed according to the directive 2010/63/EU of the EU legislation, and approved by the local authorities Behörde für Justiz und Verbraucherschutz der Freien und Hansestadt Hamburg, Department for Lebensmittelsicherheit und Veterinärwesen (protocol code N032/2020 April 22nd 2020). Eight to ten weeks old female and male golden Syrian hamsters were purchased from Janvier Labs (Le Genest-Saint-Isle, France). The hamsters were housed under standardized conditions (21 ± 2 °C, 40–50% humidity, food, and water ad libitum) with a 12:12 light–dark cycle in groups of two to four animals at the Heinrich Pette Institute, Leibniz Institute for Experimental Virology in Hamburg, Germany as previously published by Stanelle-Bertram et al. [24].

Intranasal infection either with 10^5^ plaque-forming units (p.f.u.) SARS-CoV-2 or phosphate-buffered saline (mock infection), respectively, was performed under general anesthesia (150 mg/kg ketamine and 10 mg/kg xylazine).

**Figure 1:**
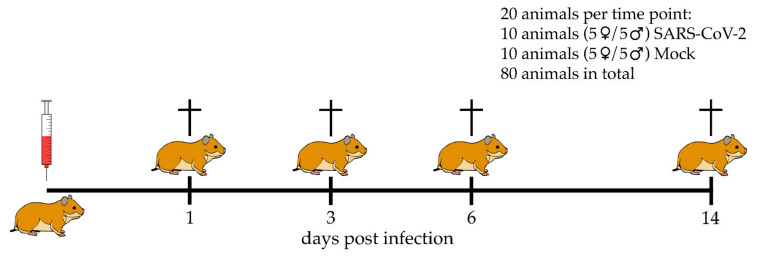
Study design. Groups of 10 golden Syrian hamsters were infected with severe acute respiratory syndrome coronavirus-2 (SARS-CoV-2) or phosphate-buffered saline (mock infection), respectively. At 1, 3, 6, and 14 days post-infection SARS-CoV-2 infected and mock-infected animals were euthanized.

At 1, 3, 6, and 14 days post-infection (dpi) ten animals per group were euthanized by intraperitoneal injection of an overdose of pentobarbital (800 mg/kg) and blood withdrawal by cardiac puncture (Figure 1). For microscopic and ultrastructural evaluation, lung tissue was collected and fixed in 10% neutral-buffered formalin (Chemie Vertrieb GmbH & Co Hannover KG, Hannover, Germany) or 5% glutaraldehyde (Merck KGaA, Darmstadt, Germany), respectively. Viral titers were evaluated at 1, 3, and 6 dpi by quantitative reverse transcription real-time PCR (qRT-PCR) as previously published by Becker et al. [23].

### 2.2. Virus

The SARS-CoV-2 strain used in this study (SARS-CoV-2/Germany/Hamburg/01/2020; ENA study PRJEB41216 and sample ERS5312751) was isolated from a human nasopharyngeal swab of a confirmed COVID-19 patient and propagated for three serial passages in Vero E6 cells using Dulbecco’s Modified Eagle’s Medium (DMEM; Sigma-Aldrich GmbH) supplemented with 2% fetal bovine serum, 1% penicillin-streptomycin and 1% L-glutamine at 37 °C. All infection experiments with SARS-CoV-2 were performed under biosafety level 3 (BSL-3) conditions at the Heinrich Pette Institute, Leibniz Institute for Experimental Virology in Hamburg, Germany [24].

### 2.3. Histochemistry

For histological examination, formalin-fixed lung samples were routinely embedded in paraffin wax. 2 µm thick serial sections were processed routinely and stained with hematoxylin and eosin (H&E). Vascular alterations were evaluated quantitatively with special emphasis on the presence of endothelial hypertrophy, endothelialitis, and vasculitis depending on the vessel diameter (<100 µm = small; 100–200 µm = medium; >200 µm = large). The numbers of affected and non-affected vessels were counted separately and are indicated as percentage of all vessels counted.

### 2.4. Immunohistochemistry

Immunohistochemistry of lung sections was performed using the avidin-biotin-peroxidase complex (ABC; Vector Laboratories, Burlingame, CA, USA) method or the Dako EnVision+ polymer system (Dako Agilent Pathology Solutions) as previously described [26,27,28]. Primary antibodies were applied overnight at 4 °C. Utilized antibodies, dilutions, detection systems, and pretreatments are detailed in Table 1.

SARS-CoV-2 nucleoprotein (SARS-CoV-2 NP) was quantified using a semiquantitative scoring system within vascular cross-sections, including tunica intima, tunica media, and tunica adventitia. The number of positive cells was assessed for each vessel individually as lack of signal, very mild (single cells), mild (≤ 25% of cells), moderate (26–50% of cells), or severe (> 50% of cells). Based on these values, a modal value (positive cell score) for each animal was determined and indicated as: no signal (0) very mild (0,5), mild (1), mild to moderate (2), moderate (3), mild to severe (4), moderate to severe (5), or severe (6). Signal distribution was then assessed as: focal/oligofocal (< 4 foci; 1), multifocal (≥4 to <15 foci; 2), diffuse (≥ 15 foci, 3). For final interpretation, an overall score for each animal was calculated as the product of positive cell score (0–6) and signal distribution score (1–3). The expression of investigated immune cell markers (CD3, T-lymphocytes; CD204, macrophages; myeleoperoxidase, neutrophils/heterophils) was scored semiquantitatively within the perivascular region, the vascular wall, and the vascular lumen of pulmonary blood vessels. Immune cell infiltration in each of the respective regions (perivascular, vascular wall, and vascular lumen) was quantified according to the scheme applied to SARS-CoV-2 NP. Final scores were calculated for each region and animal.

### 2.5. Combined Stainings

To visualize vascular integrity together with perivascular edema formation, different immunohistochemical markers (anti-AQP1; anti-CD31; anti-laminin) were combined with a modified alcian blue staining. Lung sections were incubated with primary antibodies detecting aquaporin 1 (AQP1, 1:1000, rabbit polyclonal, PA5-78806, Invitrogen, Carlsbad, CA, USA) or laminin (1:50, rabbit polyclonal, L9393, Sigma-Aldrich Chemie GmbH, Taufkirchen, Germany) overnight at 4 °C followed by the appropriate secondary antibody (Horse Anti-Rabbit IgG, biotinylated, BA-1100, Vector Laboratories, Burlingame, CA, USA) and the ABC for 30 min at room temperature. The primary antibody specific for CD31 (1:100, rabbit polyclonal, AP15436PU-M, Acris Antibodies GmbH, Herford, Germany) was applied using the EnVision+ System (Dako Agilent 425 Pathology Solutions).

After immunohistochemistry, slides were incubated for 2 min in 3% acetic acid (Carl Roth GmbH & Co., Karlsruhe, Germany) and stained for 2 h in 0.1% alcian blue solution (Chroma Gesellschaft Schmid & Co, Stuttgart, Germany) diluted in 3% acetic acid at pH 2.5. Afterward, slides were rinsed in tap water followed by a graded series of alcohol and routine mounting. Specific nuclear staining was omitted.

For interpretation of vascular integrity, vessels exhibiting edema and those without were counted. Vessels were grouped by their circumferential expression of AQP1 (0% AQP1; > 0 ≤ 25% AQP1; > 25 ≤ 50% AQP1; > 50 ≤ 99% AQP1; 100% AQP1) and subdivided into three groups, depending on their diameter (<100 µm = small; 100–200 µm = medium; >200 µm = large).

### 2.6. Transmission Electron Microscopy

Morphology and integrity of pulmonary vessels were further evaluated using transmission electron microscopy. Glutaraldehyde-fixed lung samples were rinsed in cacodylate buffer (Serva Electrophoresis GmbH, Heidelberg, Germany) for one night followed by post-fixation with 1% osmium tetroxide (Roth C. GmbH & Co. KG, Karlsruhe, Germany). After dehydration in graded series of alcohol, samples were embedded in epoxy resin as described before [29]. Ultrathin sections were cut from selected representative localizations, contrasted with uranyl acetate and lead citrate, and evaluated on a transmission electron microscope (EM 10C, Carl Zeiss Microscopy GmbH, Jena, Germany).

### 2.7. Statistical Analyses

Statistical analyses were conducted using SPSS for Windows^TM^ v. 27 (IBM^®^ SPSS^®^ Statistics, SPSS Inc., Chicago, IL, United States). Data were analyzed using the Shapiro–Wilk test. Significant differences within groups (SARS-CoV-2 vs. mock) were investigated using Mann–Whitney U tests. Significant differences between single dpi were investigated via Kruskal–Wallis tests followed by Dunn–Bonferroni post hoc testing. Edema formation and AQP1 expression were further evaluated using Spearman’s rank-order correlation. Statistical significance was accepted at *p*-values of ≤ 0.05 (*), ≤ 0.01 (**) and ≤ 0.001 (***), respectively. Graphs were designed using GraphPad Prism (GraphPad Software, San Diego, CA, USA) for Windows™.

## 3. Results

### 3.1. Characterization of Vascular Alterations

**Figure 2:**
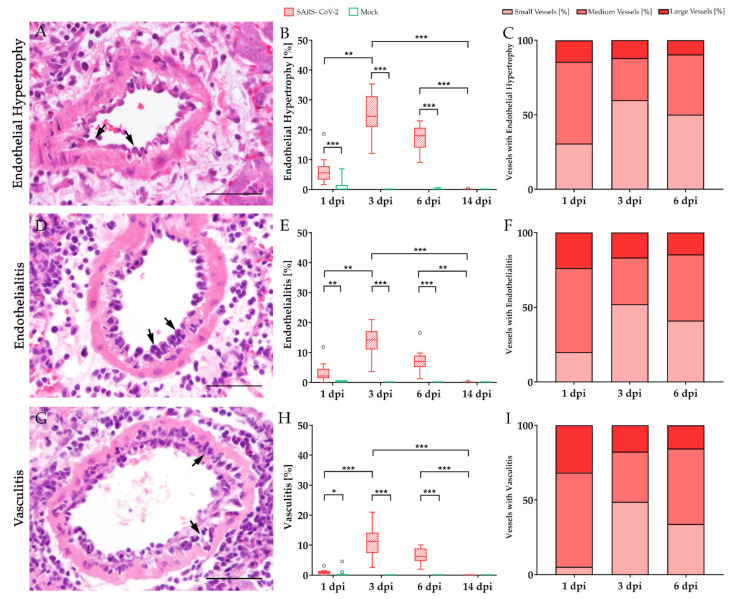
Characterization of vascular alterations in severe acute respiratory syndrome coronavirus-2 (SARS-CoV-2) infected hamster lungs. Vascular alterations including endothelial hypertrophy (**A**–**C**), endothelialitis (**D**–**F**), and vasculitis (**G**–**I**) were quantified using hematoxylin and eosin (H&E) staining. All three vascular features could be detected in SARS-CoV-2 infected animals at 1 day post-infection (dpi) and were resolved at 14 dpi. Statistical analyses revealed a significant increase of endothelial hypertrophy, endothelialitis, and vasculitis in SARS-CoV-2 infected hamsters at 3 compared to 1 dpi as well as a significant decrease in pathological alterations until 14 dpi (**B**,**E**,**H**). The stacked bar charts represent the percentage of inflamed vessels separated according to their size (**C**,**F**,**I**). Small and medium-sized vessels were especially affected in high numbers and proportions. Furthermore, the number of small vessels affected by endothelial hypertrophy, endothelialitis, and vasculitis increased from 1 dpi to 3 dpi. Data are shown as box and whisker plots with mean and quartiles or as a stacked bar chart, respectively. Significant differences between mock and infected animals obtained by Mann–Whitney U tests and between dpi obtained by Kruskal–Wallis tests, followed by Dunn–Bonferroni post hoc testing are indicated by * (* *p* ≤ 0.05, ** *p* ≤ 0.01, *** *p* ≤ 0.001). Representative images of vessels were taken at 400× magnification, bars represent 50 µm.

Characterization and quantification of pulmonary vascular alterations were performed in golden Syrian hamsters experimentally infected with SARS-CoV-2 (Figure 2). Vascular alterations included endothelial hypertrophy as well as inflammatory changes comprising endothelialitis and vasculitis. Histologically, endothelial cells frequently exhibited bulging into the vascular lumen (endothelial hypertrophy, Figure 2A arrows). Furthermore, mononuclear inflammatory cells (macrophages and lymphocytes), could be observed directly beneath or within the endothelial cell layer (endothelialitis, Figure 2D arrows). Vascular walls were enlarged by inflammatory infiltrates composed of neutrophils/heterophils, macrophages, and lymphocytes (vasculitis, Figure 2G arrows). Hypertrophic and inflammatory changes occurred both alone and in combination with each other. Endothelial hypertrophy, endothelialitis, and vasculitis could be detected as early as 1 dpi, reaching maximum values at 3 dpi and were resolved at 14 dpi. Statistical analysis revealed significant differences between SARS-CoV-2 and mock-infected animals at 1, 3, and 6 dpi. Moreover, all three parameters showed a significant increase of values between 1 and 3 dpi, followed by the complete disappearance of detectable pathological changes until 14 dpi (Figure 2B,E,H). A high proportion of small and medium-sized vessels was especially affected (Figure 2C,F,I). Only very low numbers of large vessels could be detected within tissue samples. Therefore, further analyses were focused on small and medium-sized vessels.

### 3.2. Absence of SARS-CoV-2 Antigen in Pulmonary Blood Vessels

**Figure 3:**
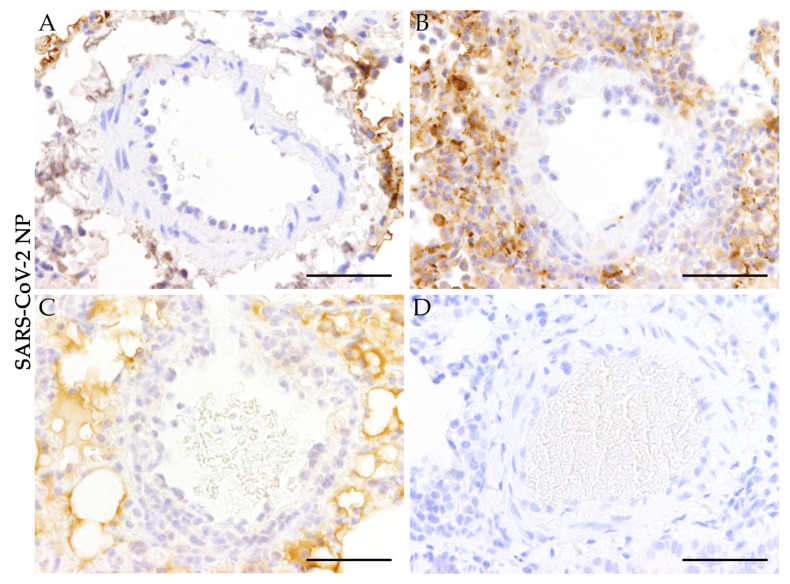
Absence of severe acute respiratory syndrome coronavirus-2 nucleoprotein (SARS-CoV-2 NP) in pulmonary blood vessels of SARS-CoV-2 infected hamsters. To investigate the presence of SARS-CoV-2 antigen in the vasculature of infected animals, a primary antibody detecting SARS-CoV-2 NP was applied. (**A**–**D**) show representative images of altered blood vessels in the lungs of SARS-CoV-2 infected animals at 1, 3, 6, and 14 days post-infection (**A** = 1 dpi; **B** = 3 dpi; **C** = 6 dpi; **D** = 14 dpi). SARS-CoV-2 antigen could not be detected in altered and non-altered blood vessels of SARS-CoV-2 infected animals. Representative images of lung tissue were taken at 400x magnification, bars represent 50 µm (**A**–**D**).

Immunohistochemical staining for SARS-CoV-2 NP was applied to evaluate the presence of SARS-CoV-2 in altered pulmonary blood vessels and reactivity was scored semiquantitatively (Figure 3). While SARS-CoV-2 antigen could be detected multifocally in the pulmonary tissue surrounding the vessels of infected animals at 1, 3, and 6 dpi (Figure 3A–C), virus antigen could not be determined within vascular tissue at any time. These results indicate that the pulmonary vascular lesions do not represent a direct effect of virus replication within vascular cells (Figure 3A–D).

### 3.3. Vascular and Perivascular Inflammation

**Figure 4:**
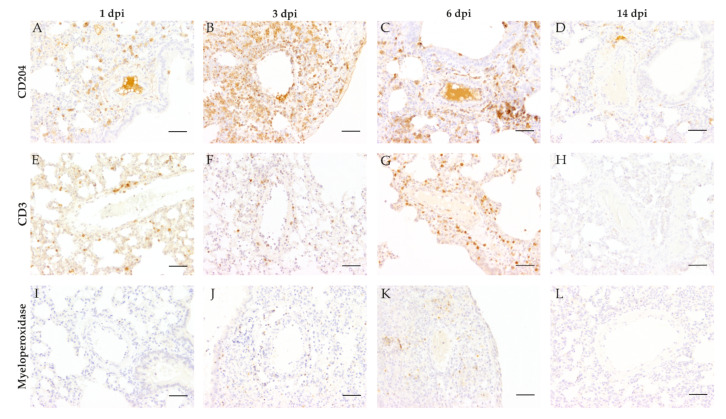
Characterization of vascular and perivascular inflammation in severe acute respiratory syndrome coronavirus-2 (SARS-CoV-2) infected hamster lungs. To further characterize the observed inflammatory vascular changes, lung tissue from SARS-CoV-2 and mock-infected animals were immunolabeled detecting intralesional macrophages (anti-CD204, **A**–**D**), T-lymphocytes (anti-CD3, **E**–**H**), and neutrophils/heterophils (anti-myeloperoxidase, **I**–**L**). CD204 positive macrophages represented the main population of intralesional immune cells, showing mild to moderate infiltration at 1 day post-infection (dpi, **A**), severe vascular and perivascular infiltration at 3 dpi (**B**), and moderate to severe infiltration at 6 dpi (**C**). T-lymphocytes showed very mild to mild infiltration at 1 (**E**) and 3 dpi (**F**) and moderate infiltration of the perivascular region and the vascular wall at 6 dpi (**G**). Very mild to mild infiltration of the vasculature by myeloperoxidase positive neutrophils/heterophils was only visible in the perivascular region at 3 (**J**) and 6 dpi (**K**), while no cells were detected at 1 dpi (**I**). At 14 dpi, all markers returned to base level detecting solely no to singular immune cells. Representative images of lung tissue of SARS-CoV-2 infected hamsters were taken at 200× magnification, bars represent 50 µm.

To further analyze the amount of infiltrating immune cell subtypes immunohistochemistry targeting macrophages (CD204), T-lymphocytes (CD3), and neutrophils/heterophils (myeloperoxidase) was performed (Figure 4). At 1, 3, and 6 dpi infiltrating immune cells were predominantly composed of CD204-positive macrophages. Inflammatory cells showed a notable infiltration into vascular walls at 1 dpi (Figure 4A), with increasing severity of vascular and perivascular infiltration until 3 dpi (Figure 4B), followed by a reduction of the inflammatory reactions at 6 dpi (Figure 4C). Appreciable numbers of T-lymphocytes were present in most animals at 3 and 6 dpi. Infiltration of T-lymphocytes was very mild to mild at 3 dpi (Figure 4F). Furthermore, moderate infiltration of the perivascular region and the vascular wall could be detected at 6 dpi (Figure 4G). Slight infiltration of the vasculature by myeloperoxidase positive neutrophils/heterophils was only detected in the perivascular region at 3 (Figure 4J) and 6 dpi (Figure 4K). No myeloperoxidase positive cells were detected at 1 dpi (Figure 4I). At 14 dpi only isolated cells stained positive for the three markers applied, indicating a receding inflammatory process.

**Figure 5:**
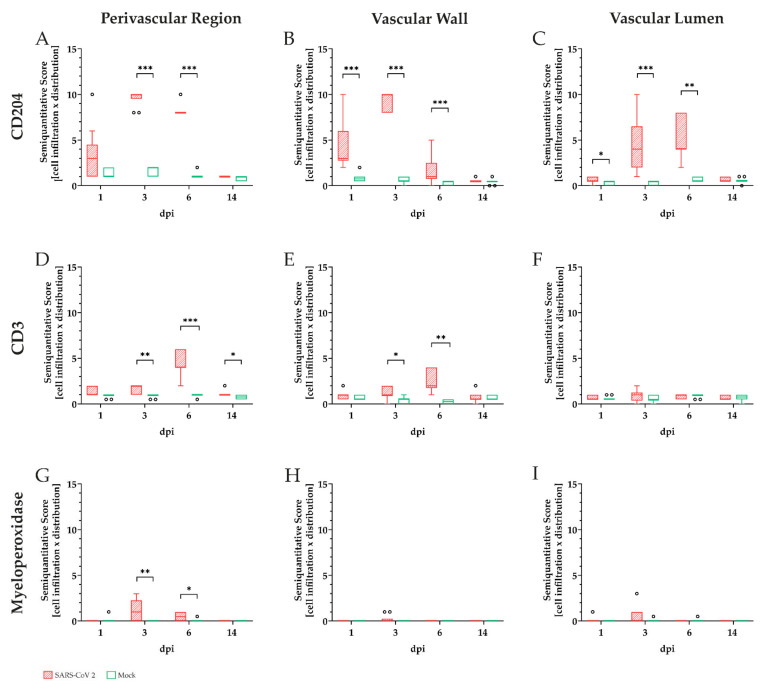
Semiquantitative analysis of inflammatory cells within different compartments (perivascular, vascular, and intraluminal) in the pulmonary vasculature of golden Syrian hamsters during severe acute respiratory syndrome coronavirus-2 (SARS-CoV-2) in comparison to mock-infected animals. Immunoreactivity of macrophages (CD204, **A**–**C**), T-lymphocytes (CD3, **D**–**F**), and neutrophils/heterophils (myeloperoxidase, **G**–**I**) was examined by light microscopy. Overall immune cell infiltration in respective areas was rated as very mild (0,5), mild (1), mild to moderate (2), moderate (3), mild to severe (4), moderate to severe (5), or severe (6). Signal distribution was assessed as: focal/oligofocal (< 4 foci; (1), multifocal (≥ 4 foci; (2) or diffuse (3). For better comparative interpretation a final score for each area and animal was calculated as the product of cell infiltration and signal distribution. Macrophages (**A**–**C**) represented the main infiltrating immune cell population in all analyzed areas, with the highest scores at 3 dpi. Statistically significant infiltration could be observed at 3 and 6 dpi in the perivascular region (**A**) and at 1, 3, and 6 dpi in the vascular wall (B). Perivascular and vascular detection of T-lymphocytes (**D**,**E**) was overall lower at all time points compared to macrophages, with significant infiltration detectable at 3, 6, and 14 dpi in the perivascular region (**D**) and at 3 and 6 dpi in the vascular wall (**E**). Significant levels of neutrophils/heterophils were mainly located perivascularly and detected at 3 and 6 dpi (**G***).* Data are shown as box and whisker plots with mean and quartiles. Significant differences between the groups obtained by the Kruskal–Wallis test followed by Dunn-Bonferroni post hoc testing are indicated by * (* *p* ≤ 0.05, ** *p* ≤ 0.01, *** *p* ≤ 0.001).

For comparative interpretation and identification of differences in distribution patterns, cellular infiltrates were scored semiquantitatively within the following regions: perivascular region, vascular wall, and vascular lumen (Figure 5). Macrophages (Figure 5A–C) represented the main infiltrating immune cell population in all analyzed areas.

Comparison of SARS-CoV-2 and mock-infected animals revealed statistically significant infiltration of all three cell types in the perivascular region at 3 and 6 dpi as well as for T-lymphocytes at 14 dpi (Figure 5A,D,G).

Significant infiltration of vascular walls by macrophages was detected at 1, 3, and 6 dpi (Figure 5B), showing maximum infiltration at 3 dpi. Significant numbers of T-lymphocytes were present at 3 and 6 dpi, whereas only low numbers of neutrophils/heterophils could be observed within the vascular walls (Figure 5E,H).

Within vascular lumen, CD204 positive macrophages again represented the predominant inflammatory cell type with significant differences between infected and mock-infected animals at 1, 3, and 6 dpi. Inflammatory cell numbers were most pronounced at 3 dpi (Figure 5C). At the end of the investigation period (14 dpi), neither the vascular wall nor the vascular lumen showed significant numbers of inflammatory cells (Figure 5B,C,E,F,H,I).

### 3.4. Aquaporin 1 Expression during Perivascular Edema

**Figure 6:**
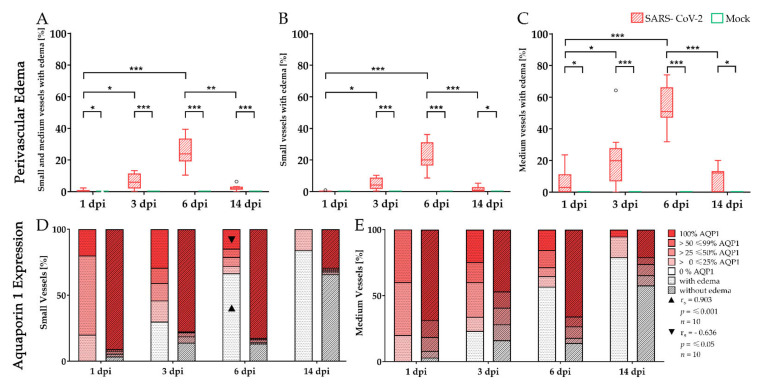
Aquaporin 1 expression during perivascular edema in severe acute respiratory syndrome coronavirus-2 (SARS-CoV-2) infected hamster lungs. Immunohistochemistry targeting aquaporin 1 (AQP1) water channels, as well as a subsequent alcian blue staining, were applied to evaluate the effects of the inflammatory vascular alterations upon vascular integrity. In SARS-CoV-2 infected animals perivascular edema could be detected in both small and medium-sized vessels at 1, 3, 6, and 14 days post-infection (dpi) compared to controls (**A**–**C**). From 1 to 6 dpi, edema affecting medium-sized vessels increased (**C**). Small vessels were also affected but to a lower degree (**B**). The stacked bar charts represent the circumferential expression of the water channel AQP1 in small (**D**) and medium-sized (**E**) vessels depending on the presence (stacked bars with dotted pattern) or absence of edema (stacked bars with striped pattern). Interestingly, vessels exhibiting edema more often reveal a decreased expression of AQP1 compared to vessels without edema. Furthermore, numbers of vessels lacking AQP1 expression increased over time with partial to complete loss of the water channel at 3, 6, and 14 dpi. Triangles indicate a strong positive relationship between AQP1 = 0% and edema formation (r_s_ = 0.903; *p* ≤ 0.001; *n* = 10) and a strong negative relationship between AQP1 = 100% and edema formation (r_s_ = −0.636; *p* ≤ 0.05; *n* = 10) using Spearman’s rank-order correlation. Data are shown as box and whisker plots with mean and quartiles or as stacked bar charts respectively. Significant differences between mock and infected animals obtained by Mann–Whitney U tests and between dpi obtained by Kruskal–Wallis tests, followed by Dunn–Bonferroni post hoc testing are indicated by *, *p* ≤ 0.05 (* *p* ≤ 0.05, ** *p* ≤ 0.01, *** *p* ≤ 0.001).

Immunohistochemistry targeting AQP1 water channels were combined with subsequent alcian blue staining to evaluate the consequences of the observed inflammatory vascular alterations in SARS-CoV-2 infected hamster lungs (Figure 6). As small and medium-sized vessels accounted for the largest proportion of vascular alterations, these two vessel calibers were evaluated together (Figure 6A) and separately (small-sized vessels, Figure 6B; medium-sized vessels, Figure 6C). Statistical analysis of the alcian blue stained small and medium-sized vessels revealed the formation of perivascular edema in SARS-CoV-2 infected lungs at 1, 3, 6, and 14 dpi compared to mock-infected controls (Figure 6A). The percentage of affected small and medium-sized vessels increased from 1 to 6 dpi, peaked at 6 dpi, and almost vanished until 14 dpi (Figure 6A). In addition, medium-sized vessels were more frequently affected by perivascular edema than small-sized vessels (Figure 6B,C).

To investigate the relation of vascular integrity and the observed perivascular edema in SARS-CoV-2 infected animals, small and medium-sized vessels were evaluated by their circumferential expression of AQP1 (Figure 6D,E). Therefore, vessels with edema (stacked bars with dotted pattern) were compared to vessels without edema (stacked bars with striped pattern). Small and medium-sized vessels with edema showed less frequently a complete and circumferential expression of AQP1 compared to the vessels without edema. Furthermore, at 3, 6, and 14 dpi a complete loss of aquaporin was especially observed in vessels with edema, with increasing numbers of affected vessels over time. Statistical analysis revealed a positive relationship between the formation of edema and the loss of AQP1 water channels in small vessels at 6 dpi with a correlation coefficient of 0.903. At the same time point, a negative correlation coefficient of −0.636 between the expression of AQP1 and the presence of perivascular edema could be observed in small-sized vessels, indicating an interdependency between loss of AQP1 and edema formation in SARS-CoV-2 infected animals.

### 3.5. Integrity of Basement Membrane and Vascular Endothelium

**Figure 7:**
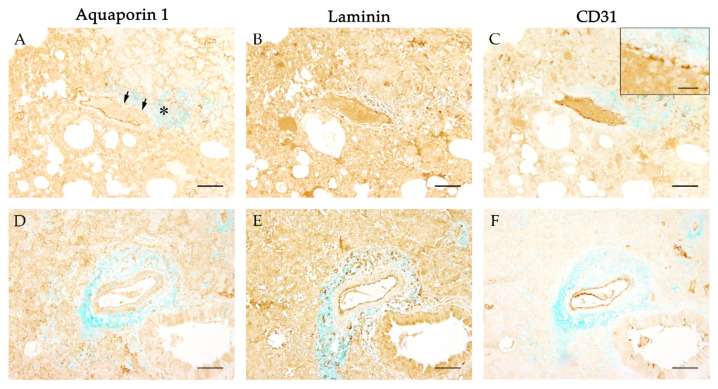
Evaluation of the basement membrane and endothelial integrity during severe acute respiratory syndrome coronavirus-2 (SARS-CoV-2) induced perivascular edema. To evaluate the integrity of the basement membrane aquaporin 1 (AQP1) immunostaining (**A**,**D**) was compared with immunohistochemistry targeting laminin (**B**,**E**). The vascular endothelium was visualized using immunohistochemistry detecting CD31 (**C**,**F**). To investigate the primary antibodies with the perivascular edema in a comparative manner, an alcian blue counterstaining was applied additionally. While vascular leakage (**A**, asterisk) was associated with a segmental (**A**, arrows) or even complete circumferential loss (**D**) of the water channel AQP1, the same vessels showed continuous labeling for laminin (**B**,**E**) and CD31 (**C**,**F**). Occasionally the circumferential expression of CD31 appeared segmentally disrupted (**C**, insert). Representative images of vessels were taken at 100x magnification, bars represent 100 µm in overviews and 20 µm in the insert.

To further analyze a possible link between loss of vascular integrity and the emergence of perivascular edema, immunohistochemistry targeting components of basement membrane (laminin) and vascular endothelium (CD31) was combined with alcian blue staining (Figure 7). Vessels revealed a regular and circumferential expression of laminin indicating an intact basement membrane (Figure 7B,E), and circumferential labeling with CD31 was detected as well (Figure 7C,F). However, a segmental disruption and irregularity of the CD31 expression could be observed occasionally (Figure 7C, insert).

### 3.6. Ultrastructural Evaluation of Vascular Alterations in SARS-CoV-2 Infected Lungs

**Figure 8:**
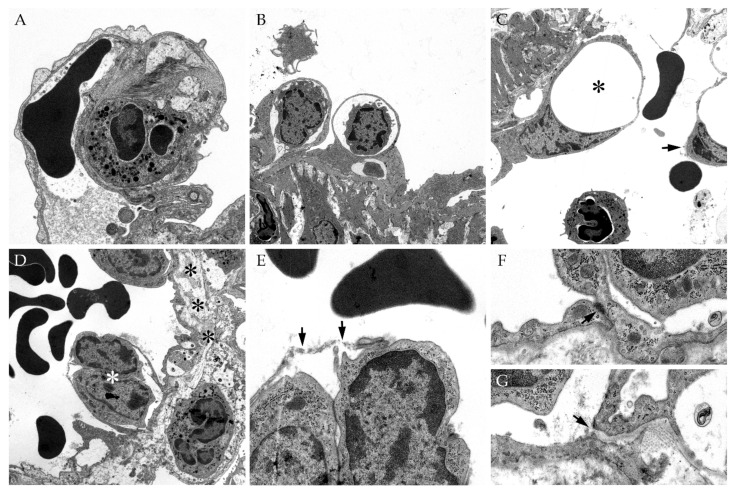
Ultrastructural evaluation of pulmonary vessels in severe acute respiratory syndrome coronavirus-2 (SARS-CoV-2) infected hamster lungs. Transmission electron microscopy of SARS-CoV-2 infected hamster lungs revealed severe vascular alterations starting at 3 days post-infection (dpi). At 3 dpi, neutrophils/heterophils could be observed within the wall of small capillaries (**A**). In SARS-CoV-2 infected lungs, inflammatory cells were also present in small cavities within the vascular endothelium (**B**) leading to endothelialitis, vacuolization (**C**, asterisk), or even complete detachment (**C**, arrow) of endothelial cells from the basement membrane. Furthermore, severe edema could be observed distending the underlying interstitium (**D**, black asterisks). Pictures (**E**–**G**) show a higher magnification of the endothelialitis in picture (**D**) (white asterisk). During the SARS-CoV-2 infection, the endothelial membranes were disrupted multifocally (**E**, arrows) and exhibited loss of intercellular junctions (**F**,**G**, arrows). Magnifications are 8.000× in (**A**), 3.150× in (**B**), 4.000× in (**C**,**D**), 12.500 in (**E**), and 20,000 in (**F**,**G**).

Transmission electron microscopy of SARS-CoV-2 infected hamster lungs was performed to evaluate the vascular integrity on the ultrastructural level and to substantiate the findings observed in immunohistochemistry (Figure 8). Representative vascular alterations in SARS-CoV-2 infected animals could be observed at 3 dpi including infiltration of capillary walls and vascular endothelium by inflammatory cells (Figure 8A,B). Furthermore, a vacuolization and detachment of affected endothelial cells were present (Figure 8C, asterisk and arrow). Edema formation with subsequent distension of the underlying interstitium could be observed as well (Figure 8D, black asterisks). These ultrastructural findings in SARS-CoV-2 infected lungs could be attributed to a disruption of the endothelial membrane and a loss of intercellular junctions (Figure 8E,F,G).

## 4. Discussion

Pulmonary vascular alterations represent a specific feature of SARS-CoV-2 infection and finally result in a breakdown of the blood–air barrier with subsequent perivascular edema formation [2,5]. However, the observed vascular alterations lack further characterization, and underlying mechanisms leading to vascular barrier breakdown remain incompletely understood. The aim of the present study was to characterize the vasculopathy during SARS-CoV-2 infection and to evaluate vascular integrity regarding inflammatory and ultrastructural alterations using the golden Syrian hamster model [30].

This model has already been used within various SARS-CoV-2 studies [9,19,20,21,22,23,24,25]. These previously published studies emphasized several significant similarities to lesions observed in SARS-CoV-2 infected patients including histopathological patterns of pulmonary alterations [30]. Furthermore, the golden Syrian hamster represents a naturally susceptible and more practical animal model compared to primates [30]. The present study revealed that vascular alterations were mainly composed of endothelial hypertrophy, endothelialitis, and vasculitis. Similar histopathological pulmonary alterations have been described in multiple post mortem studies of COVID-19 infected patients reporting vasculitis, endothelialitis, and perivascular inflammation [8,31,32,33,34]. In human medicine, virus detection using RT-PCR represents the standard method to ascertain COVID-19 infection in humans [33]. PCR solely detects viral ribonucleic acid (RNA). However, it does not provide any information about replication or the exact presence or absence of virus within lesion sites. Therefore, these methods cannot clarify a potential causative relationship between the virus infection, lesion development, and finally a person’s demise [33,35]. Immunohistochemical detection of SARS-CoV-2 antigen provides a suitable approach to overcome these limitations, especially by showing the exact location of virus antigen within infected tissue. Moreover, it helps to exclude false-positive as well as false-negative PCR results underlining the importance also in human post mortem examinations [33]. In SARS-CoV-2 infected hamsters, vascular alterations were most prominent at 3 and 6 dpi. At these days, vasculopathy especially affected small and medium-sized vessels, resembling vascular alterations described for the Kawasaki-like syndrome of patients suffering from SARS-CoV-2 infection [36,37].

Hitherto, several underlying mechanisms leading to vascular leakage and edema as observed in patients with severe COVID-19 infection have already been discussed [2]. First, recruitment of inflammatory cells, either by a primary viral infection or immune-mediated, induces diffuse endothelial inflammation, dysfunction, and apoptosis [2,5,6]. Secondly, the virus binds to angiotensin-converting enzyme 2 (ACE2) receptors, lowers ACE activity, and activates the kallikrein–bradykinin pathway leading to further vascular leakage [2,38,39]. Thirdly, an overwhelming immune response described as a “cytokine storm” activates reactive oxygen species (ROS) and contributes to an elevated consumption of the vasodilation regulator nitric oxide (NO) [2,40]. Finally, the exaggerated cytokine expression and the secretion of vasoactive molecules such as thrombin, histamine, bradykinin, thromboxane A2, and vascular endothelial growth factor (VEGF) result in a disruption of the vascular endothelium [2]. In addition, extracellular traps (ETs) released by neutrophils/heterophils and macrophages contribute to vessel inflammation or endothelial damage and have been identified as a potential driver of COVID-19 [5,8,41,42,43,44]. However, the relationship between the presence of virus and associated immune response at the cellular level remains poorly defined [45].

Interestingly, despite the high similarity of vascular lesions to human lungs, golden Syrian hamsters lack the expression of ACE2 receptors in endothelial and smooth muscle cells of the vessel wall [21]. Furthermore, previous studies could not detect SARS-CoV-2 within vascular lesions at the RNA level [9]. The present study substantiated this observation on the virus protein level, indicating that vascular lesions are not the consequence of direct viral effects upon the pulmonary vasculature. Therefore, vascular lesions most likely represent the consequence of immune-mediated effects caused by infiltrating immune cells.

In this study, macrophages represent the predominant inflammatory cell type with the highest observable numbers at 3 dpi. Although the participation of neutrophils/heterophils and lymphocytes has been reported in previous studies, the present study reveals a dominant presence of macrophages indicating an important role in the development and progression of vascular lesions during SARS-CoV-2 infection [5,9]. In addition to their capacity for phagocytosis and antigen presentation, macrophages are able to produce and release a broad spectrum of host defense-relevant mediators and inflammatory cytokines. The macrophage-derived cytokine tumor necrosis factor (TNF) is able to remodel endothelial intercellular junctions allowing the transmigration of additional leukocytes [46,47,48,49]. Furthermore, the release of ROS and matrix metalloproteinases could directly lead to damage of vascular integrity [49,50,51,52,53,54,55]. The potential role of ETs released either directly by the invading macrophages or surrounding neutrophils/heterophils should be considered as an additional driver of vascular damage [5,8,41,42,43,44,56].

To evaluate the consequences of the observed vascular alterations and the resulting leakage, alcian blue staining was performed. At all investigated time points SARS-CoV-2 infected animals exhibited a perivascular edema as demonstrated by a prominent alcian blue staining. While inflammatory cell infiltration was most pronounced at 3 dpi, perivascular edema formation reached its maximum at 6 dpi. This temporal sequence again strengthens the assumption that vascular damage is initiated by the inflammatory cell infiltration. Vascular leakage as a result of inflammatory cell infiltration was as well described for SARS-CoV-2 infected patients, though the underlying pathologic mechanisms leading to perivascular edema in human lungs need to be investigated [5,8].

Affected vessels showed a strong and circumferential expression of laminin, confirming the preserved integrity of the basement membrane complex as described for other forms of vascular edema [57]. However, the segmental irregular expression of CD31 indicated a disruption of the endothelial cell layer. Fenestration of the endothelial barrier with an increase of vascular permeability is reported to be the result of the rearrangement and loss of intercellular tight and adherens junctions, resulting in gap formation [11,58].

Previous studies already discussed the role of AQP1 in the intercellular adhesion of endothelial cells [10,11,59]. Therefore, immunohistochemistry targeting AQP1 was combined with the alcian blue staining visualizing the perivascular edema. Interestingly, the formation of perivascular edema was associated with the loss of AQP1 water channels. These findings were accompanied by a loosening of intercellular junctions partly resulting in complete detachment of the endothelial cells, observed ultrastructurally. The loosened cellular integrity accompanied by cell swelling, necrosis, and cellular detachment from the basement membrane is already described in both SARS-CoV-2 hamster models and human COVID-19 infections [5,9]. However, the underlying mechanism leading to the vascular leakage of fluid remains elusive.

AQP1 is not only responsible for transcellular water transport, it also controls paracellular fluid exchange. While the transcellular pathway provides transport for molecules with a radius greater than 3 nm, solutes smaller than 3 nm are transported via the paracellular pathway [10]. In addition, a connection between AQP1 and the cytoskeletal arrangement has been reported [10,59]. In the present study, the segmental or even circumferential loss of AQP1 was associated with an ultrastructural loss of integrity of the vascular endothelium and may have resulted in a loosening of intercellular junctions leading to the paracellular leakage of fluid. Infiltration of macrophages in inflammatory changes is known to affect intercellular junctions and might also be causing the observed loss of AQP1 in this study. To date, the immune-mediated mechanisms leading to a downregulation of AQP1 in pulmonary inflammation have not been fully elucidated. Studies of acute respiratory distress syndrome in domestic animals postulate a correlation between the decrease of AQP1 water channels and an increase of macrophage-derived mediators like ROS or TNF, the latter facilitating the secretion of additional cytokines such as interleukin (IL)-1A, IL-6, and IL-10 [16,60]. An upregulation of proinflammatory cytokines including TNF, IL-6, and IL-10 indicates the activation of a specific immune pathway probably leading to a decrease of AQP1 in patients suffering from COVID-19 [32,34].

Despite known limitations, for example, the lack of ACE2 receptors in hamster endothelial cells and the absence of virus protein in vessel walls reported here, the SARS-CoV-2 hamster model offers the opportunity to investigate important basic pathological changes in pulmonary damage following SARS-CoV-2 infection. These pathological principles might be harnessed for future detection of suitable pharmacological targets to overcome the limitations of animal models and to serve as a more effective treatment of severe COVID-19 cases.

## 5. Conclusions

The present study showed that SARS-CoV-2 infection leads to vascular alterations including endothelial hypertrophy, vasculitis, endothelialitis, and associated vascular leakage. The inflammatory infiltrates were mainly composed of macrophages with smaller amounts of T-lymphocytes and neutrophils/heterophils. Further analyses revealed that inflammatory vascular changes resulted in the formation of perivascular edema accompanied by the loss of AQP1 water channels. The circumferential loss of AQP1 was associated with a disruption of vascular endothelium and may have contributed to the loss of intercellular junctions leading to the formation of perivascular edema. The possible specific effect of macrophages and their secretion of proinflammatory mediators on AQP1 expression and function needs to be investigated in more detail in future studies.

## Figures and Tables

**Table 1:** Primary antibodies, pretreatment, detection systems and dilutions used for immunohistochemistry.

Antigen	Pretreatment	Dilution	Clonality	Detection System	Supplier	Catalog Number
**AQP1**	microwave/citrate buffer	1:1000	polyclonal rabbit	ABC	Invitrogen, Carlsbad, California, USA	PA5-78806
**CD3**	microwave/citrate buffer	1:800	polyclonal rabbit	ABC	Dako Agilent Pathology Solutions, Santa Clara, USA	A0452
**CD204**	microwave/citrate buffer	1:500	monoclonal mouse	ABC	Abnova Corporation, Taipeh, Taiwan	MAB1710
**CD31**	microwave/citrate buffer	1:100	polyclonal rabbit	EnVision+	Acris Antibodies GmbH, Herford, Germany	AP15436PU-M
**Laminin**	microwave/citrate buffer	1:50	polyclonal rabbit	ABC	Sigma-Aldrich Chemie GmbH, Taufkirchen, Germany	550390
**Myeloperoxidase**	microwave/citrate buffer	1:200	polyclonal rabbit	EnVision+	Abcam plc, Cambridge, United Kingdom	ab9535
**SARS-CoV-2 NP**	microwave/citrate-Na_2_H_2_EDTA buffer	1:32000	monoclonal mouse	EnVision+	Sino Biological, Peking, China	40143-MM05

## Data Availability

The data presented in this study are not publicly available but are available upon reasonable request.
